# Surgical Management of Locally Recurrent Rectal Cancer

**DOI:** 10.1155/2012/464380

**Published:** 2012-06-03

**Authors:** Niamh M. Hogan, Myles R. Joyce

**Affiliations:** ^1^Discipline of Surgery, National University of Ireland, Galway, Ireland; ^2^Department of Colorectal Surgery, University College Hospital, Galway, Ireland

## Abstract

Developments in chemotherapeutic strategies and surgical technique have led to improved loco regional control of rectal cancer and a decrease in recurrence rates over time. However, locally recurrent rectal cancer continues to present considerable technical challenges and results in significant morbidity and mortality. Surgery remains the only therapy with curative potential. Despite a hostile intra-operative environment, with meticulous pre-operative planning and judicious patient selection, safe surgery is feasible. The potential benefit of new techniques such as intra-operative radiotherapy and high intensity focussed ultrasonography has yet to be thoroughly investigated. The future lies in identification of predictors of recurrence, development of schematic clinical algorithms to allow standardised surgical technique and further research into genotyping platforms to allow individualisation of therapy. This review highlights important aspects of pre-operative planning, intra-operative tips and future strategies, focussing on a multimodal multidisciplinary approach.

## 1. Introduction

Local recurrence of rectal cancer is difficult to treat, may cause severe disabling symptoms, and often holds a dismal prognosis. Surgery remains the cornerstone of management for the majority of primary rectal cancers. Despite a marked improvement in local control with the popularisation of Total Mesorectal Excision (TME) and the use of improved chemoradiotherapeutic regimen, recurrence continues to present a significant clinical problem. Refinements in management have, however, led to a decrease in locoregional rectal cancer recurrence rates from 25–40% to 4–8% [1]. Since 20% to 50% of these patients has local recurrence in the absence of distant metastasis, it is intuitive that surgical management represents a viable treatment option [2]. Surgery for locally recurrent rectal cancer, however, requires the undertaking of complex techniques in a hostile operative environment and in many cases requires input from other specialities such as urology, gynaecology and vascular teams. These surgeries should in principle only be performed in a tertiary centre with appropriate surgical, anaesthesiology, and intensive care expertise. Postoperative morbidity is high, ranging from 15–70% and increases with the complexity of resection performed [3, 4]. As a result, surgical management of local recurrence of colorectal cancer has not attained the international approval which has been bestowed upon resection of distant metastases such as hepatic disease. Despite many potential pitfalls, surgery remains the only therapy with curative potential and safe surgery is feasible. This paper highlights pertinent issues regarding surgical preparation and techniques with a focus on the importance of a multimodal approach.

## 2. Mode of Presentation and Risk Factors

 In 70% of cases, recurrence of rectal cancer occurs within two years of primary surgery, while 85% occurs within 3 years [4, 5]. Mode of presentation is varied and may be dependent on the site of disease. Up to one-third of patients does not present with any symptoms [4, 5], emphasising the importance of a carefully designed and diligent schedule of postoperative followup. A recent population-based cohort study of 57 patients concluded that followup after rectal cancer surgery by annual clinical examination is not sufficient. They reported that at diagnosis of local recurrence 86% of patients was symptomatic and 70% was diagnosed between scheduled follow-up visits [6]. The nature of this schedule is dependent on the type of primary resection performed. After sphincter-preserving surgery, surveillance to facilitate early diagnosis of recurrence should comprise digital rectal examination, sigmoidoscopy, and enquiry regarding symptoms of bleeding or changes in bowel habit. In contrast, the majority of local recurrences after abdominoperineal resections are diagnosed after detection of elevated CEA levels or upon report of pelvic pain [7]. When present, symptoms tend to be disabling and persistent. Refractory pelvic pain, tenesmus and malodorous discharge are common [8] and quality of life is often detrimentally affected [9, 10]. Pain on presentation has been identified as a significant predictor of inferior long-term survival [11, 12]. This is likely related to the association between extent of pain and degree of fixation in the pelvis, reflecting a more advanced stage of local recurrence at presentation and therefore worse prognosis.

Several risk factors are reported to be associated with local recurrence. These may be broadly grouped into pathological, anatomic, and surgical factors. Degree of lymphovascular invasion, differentiation, and tumour size has been associated with increased risk of local recurrence [13]. Anatomically, positive circumferential or distal resection margin at initial resection, including positive microscopic margins, increases risk. In patients who have received neoadjuvant chemoradiation, a margin of less than 1 cm is considered oncologically adequate [14]. Patients operated on in high-volume centres have also been reported to enjoy lower recurrence rates [15] and surgical technique may also play a role. In selected series, abdominoperineal resection (APR) has been associated with higher recurrence rates than sphincter-preserving surgery [16]. In addition, newer lower excision techniques such as transanal endoscopic microsurgery (TEMS) may also increase risk and patients should be carefully selected since recurrence rates are increased according to stage. A retrospective analysis of 74 patients with T1 and T2 rectal adenocarcinoma treated with TEMS and 100 patients with T1N0M0 and T2N0M0 rectal adenocarcinoma treated with radical surgery showed a statistical difference in 5-year local recurrence rates for T2 but not T1 cancers [17]. Elevation in serum CEA lacks sensitivity (59%) but has a specificity of 84% [18]. Recent efforts towards identifying novels biomarkers to predict recurrence in colorectal cancer have shown early promise [19]; however, further investigation is necessary.

## 3. Anatomical Classification

Although TME has contributed dramatically to improved management of primary rectal cancer, its popularisation decreases the likelihood that a recurrent neoplasm will remain confined to a specific compartment due to the absence of visceral rectal fascia [20]. Locally recurrent rectal cancer is generally grouped according to anatomic location. An alternative system, used at the Mayo Clinic, classified these tumours according to the presence of symptoms, with a particular focus on pain, as well as degree of fixation. Although the anatomical system may be imperfect, it is currently the most widely accepted method of classification. Due to the fact that surgical approach is largely dictated by the location of recurrence and relationship to surrounding structures, the use of an anatomical classification system is practical in this setting. Axial recurrences are confined to the pelvic organs without invading into bone or sidewall. This includes anastomotic recurrence after low anterior resection (LAR), recurrence after local excision procedures, such as, TEMS and perineal recurrence after APR [21]. Tumours in the presacral space which invade into the sacrum are grouped as sacral or posterior recurrences. Anterior recurrences may involve genitourinary organs. Sidewall or lateral recurrence is diagnosed when tumour invades iliac vessels, pelvic autonomic nerves, pelvic ureters or extends through the greater sciatic foramen [22]. A growing body of evidence shows prognosis varies according to site of recurrence. Moore et al. reported that lateral or sidewall recurrences were less likely to be curatively resected than axial or anterior [14].

## 4. Surgical Management: Preoperative Preparation

Without intervention, prognosis of recurrent rectal cancer is dismal with median survival typically 6-7 months [8, 23]. These patients endure symptoms which are catastrophic to quality of life including refractory pain, discharge, and tenesmus. Only 30% of patients achieve symptom control with radiotherapy alone and this treatment option rarely improves survival beyond one year [24]. Radical surgery offers the only hope of complete therapy and up to 50% of cases is confined to the pelvis and thereby labelled theoretically amenable to cure [25]. Additionally, in carefully selected patients, surgery may be of benefit even in the presence of distant metastases with metastatsectomy gaining favour [24]. Morbidity and mortality rates of radical surgery for recurrences are high and can reach 60% and 8% (at 3 months), respectively [5].

Surgery for recurrent rectal cancer is a challenging undertaking which should ideally be individualized and performed in a specialist unit with early involvement of a multidisciplinary team. A recent systematic review reported that the proportion of potentially curative resections has increased in recent years, probably due to improved staging, neoadjuvant treatment, and increased surgical experience in dedicated centres, which has resulted in improved survival [26]. Resections of this nature, however, remain vulnerable to complication and the operative environment is often hostile. Normal tissue planes are frequently obliterated, tissues may be friable from previous irradiation, dense adhesions are often present, and fibrosis may be extensive. Unexpected discovery of previously undiagnosed peritoneal or visceral metastases is not uncommon and is a poor prognostic indicator. As a result, as much information as possible should be gathered preoperatively and communication with the patient regarding inherent risks is crucial. A specialist colorectal nurse should be involved at an early stage as a link between the patient and the lead clinician. A systematic approach is optimum and some guidance can be found in the literature. Bouchard and Efron recommend a full blood panel including carcinoembryonic antigen testing as well as thorough physical examination [21]. Where necessary this should be supplemented by digital rectal examination, vaginal examination, sigmoidoscopy, cystoscopy, or examination under anaesthesia. Full details of previous surgeries should be sought if not performed in the same centre. Mirnezami et al. advocate early assembly of a multidisciplinary which may include orthopaedic, urologic, gynaecologic, and vascular surgeons as well as colorectal specialists [24]. Plastic surgeons may also be required as recent technical improvements in reconstructive options have contributed significantly to outcome and quality of life.

Thorough preoperative staging is crucial to optimum planning and determination of resectability. Mirnezami et al. provide an excellent algorithm for initial approach to surgically resectable recurrent rectal cancer [24]. Computerized Tomography (CT) can be used to confirm the presence of a mass and investigate the presence of distant metastases. If a distant lesion is identified or if occult tumour or metastases are suspected, Positron Emission Tomography (PET) with fluorodeoxyglucose (FDG) may provide useful information to establish a diagnosis and to assess the location and metabolic activity. The ability of Magnetic Resonance Imaging (MRI) to differentiate soft tissue contrast resolution makes it useful in assessing the precise site of the tumour including relationship to vessels. Both MRI and CT demonstrate low sensitivity in accurate assessment of side wall involvement [21]. With MRI the danger of false-positive readings in patients who have received recent radiotherapy remains an issue and differentiation between fibrosis and malignant tissues is not definitive. A recent retrospective study assessing the accuracy of preoperative magnetic resonance (MR) imaging for identification of tumour invasion into pelvic structures in 40 consecutive patients found that MRI had a negative predictive value of 93%–100%. Interestingly, assessment failures were mainly because of misinterpretation of diffuse fibrosis, especially at the pelvic side walls [27]. For this reason, it is crucial to procure tissue for histological confirmation of the recurrence where possible either by colonoscopy-or CT-guided biopsy. In cases where this is not possible, a detailed and frank patient discussion is imperative, conveying the high-risk nature of surgery versus uncertainty regarding the diagnosis. If patient or surgeon is reluctant to proceed, watchful waiting is an alternative course of action [24].

## 5. Surgical Management: Timing, Contraindications, and Resectability

Determination of resectability should not only assess anatomic feasibility of performing an R0 resection but also ability to attain an acceptable level of morbidity and mortality. Careful patient selection is crucial and when surgery is planned, rigorous preoperative assessment of fitness is necessary. High patient comorbidity load often represents the first contraindication to surgery. If physical examination reveals lower limb oedema, a cause should be sought as lymphatic or venous obstruction represents an absolute contraindication to surgery. Other anatomic factors identified in preoperative imaging, such as, encasement of iliac vessels, bilateral ureteric obstruction of circumferential involvement of the pelvic wall. This is because ability to obtain negative margins is significantly compromised by involvement of these structures [14]. Tumours which involve the ureters or iliac vessels may also be associated with bony involvement at S1 and S2 level. Sacral invasion above the S2-S3 junction will almost invariably require the patient to undergo internal fixation due to sacroiliac instability. Relative and absolute contraindications are the subject of some controversy in the literature with many small studies reporting conflicting results. Henry et al., for example, conducted a retrospective analysis of single-centre experience and concluded that hydronephrosis should not constitute an independent contraindication to attempted curative resection [28]. Maslekar et al. also recently reported resection of recurrence with encasement of external iliac vessels [29]. With advances in surgical technique it is likely that in the future more and more contraindications may move from being categorised as absolute to relative.

If the patient is radiotherapy naive, at our centre, we administer long-course chemoradiotherapy (40–50 Gy) to improve local control and potential for curability. The timing of surgery may then be dictated by a 6–8 week wait after treatment followed by thorough restaging [30]. It is well recognised that the normal tissue tolerance of the intestine is often the dose-limiting factor in the administration of pelvic and abdominal radiotherapy.

## 6. Surgical Management: General Principles

Having completed the extensive preoperative phase, some general principles apply to operative approach. Aids such as ureteric stents, radiological tattooing of surface markings for the level of sacrectomy, and preoperative marking of stoma site may be helpful [24]. A multimodal strategy has gained favour in the recent past. If intraoperative radiotherapy is planned certain infrastructure is required, such as, a specialised table to enable patient positioning and optimum delivery. Generally, the patient is first put in the Lloyd-Davies position but may need to be moved to the prone position if sacral resection is planned. Exposure is critical and in our practice we generally make an incision from the xiphisternum to symphysis pubis. Wound protectors may be used to reduce potential for infection and deposit of tumour cells in the wound. Many surgeons favour a Bookwalter retractor for exposure. Complete mobilization of the small intestine is advised to rule out the presence of metastases. This phase may be time consuming due to the potential requirement for extensive adhesiolysis and the small bowel is often adherent within the pelvis. If a small bowel segment is adherent to the pelvic malignancy then it must be removed en-bloc. If ascitic fluid is present a sample is sent for cytology. In commencing pelvic dissection, Bouchard and Efron recommend beginning in a plane free of adhesions in an area away from the tumour where possible [21]. Intraoperatively, as with imaging, difficulties may arise in accurately distinguishing tumour from radiotherapy-related fibrosis. This conflict limits the use of less radical approaches significantly. There may a role for the use of frozen section intraoperatively if this helps in the decision to proceed to a more aggressive dissection versus conservative management. In many cases it may take several hours of exploration of the various planes before one can be confident that resection is feasible.

The decision regarding how to proceed with central or axial recurrences is heavily related to the involvement of urogenital organs and the primary procedure previously performed. In the case of involvement of urogenital organs, curative resection requires an en-bloc-extended radical approach according to the patient's gender. If the dome of the bladder alone is involved, a partial cystectomy will generally suffice. If the trigone is involved, and the prostate in males, total pelvic exenteration and en-bloc prostatectomy are the only curative option. In addition to MRI, cystoscopy performed prior to definitive surgery will help with surgical planning and patient consent, according to area of bladder involved. In females, involvement of the uterus or vagina requires hysterectomy. Reconstructive options, such as, an ileal conduit, colonic conduit, or vaginal reconstruction are possible. In the absence of urogenital involvement, the patient's primary procedure dictates management. In a patient who has undergone a previous anterior resection, Mirnezami and Sagar recommend radical resection outside the original plane of dissection [22]. If the primary surgery was an abdominoperineal resection (APR), a pelvic recurrence can be treated with resection of the mass and involved small bowel where necessary. With perineal recurrence, a transperineal approach may be possible and a posterior distal sacrectomy may be required. In the case of previous APR, the empty pelvis often contains involved loops of small bowel which must be resected en-bloc with the mass. The ensuing perineal defect will generally require a rectus abdominus or gracilis flap. There is currently no guidance in the literature regarding the extent of lymphadenectomy required and equipoise on this issue cannot be reached in the absence of a further clinical trials.

Presacral venous haemorrhage may be extensive, difficult to control with conventional haemostatic agents and potentially life threatening. Before embarking on a resection in this area, provisions should be made for the potential requirement of blood products, aggressive fluid resuscitation, synthetic haemostatic agents, and devices, such as, thumbtacks [31]. Sacral recurrence is best managed via two-stage-combined abdominosacral approach [22]. Dissection in the presacral plane is necessary until the mass is reached. If neural and vascular involvement is absent, limited, or confirmed to be compatible with resectability, the patient may be moved to the prone position, allowing good exposure If the tumour invades the sacrum at the S1 or S2 level we would deem this unresectable. Some centres may consider resection with subsequent internal fixation, but we believe that ensuing deterioration in quality of life could not justify the risk to benefit ratio. If the tumour is distal to S2 then a distal sacrectomy may be performed with en-bloc resection of the previously formed neorectum or mass. Stomas and ileal conduits are constructed and omentoplasty may be undertaken to fill the pelvis. This reduces the potential for small bowel to become adherent to the raw pelvic surface causing obstruction and reduces the potential for perineal wound breakdown. The more extensive the sacrectomy performed, the worse are the morbidity, mortality, and quality of life. After less extensive sacral amputation, some series report a more acceptable quality of life despite stomas and temporary pain owing to the resection of sacral nerves [32]. A recent small series from the Mayo Clinic reported promising results from high sacrectomy indicating that these surgeries may be safely performed in centres of excellence [33]. Despite a median operative time of 13.7 hours and median operative blood transfusion of 3.7 litres, thirty-day mortality was nil. The overall median survival was 31 months (range 2–39 months), and all deaths were due to metastatic disease. Although only nine patients were included in this study, similar reports are emerging from other centres [34] and potentially indicate that high sacrectomy that achieves clear margins in patients with recurrent rectal cancer is feasible. Primary closure of the skin and fat is vulnerable to wound complications, and therefore a myocutaneous flap using the rectus abdominus or the gluteal muscles may be employed [26, 35].

The group associated with worst prognosis and resectability potential is the group involving the lateral pelvic sidewall [20]. A recent review reported that the more widespread use of TME has increased the incidence of pelvic sidewall recurrence [26]. Extensive involvement is a relative contraindication to operative intervention as key structures such as the ureters, iliac vessels, and sciatic nerve may be involved. Resection of the iliac vessels is associated with significant bleeding and as discussed, preoperative stenting of the ureters is advisable to facilitate dissection and identification of the ureters during the first phase of the surgery which should begin at the pelvic brim [36]. Early control of vessels and other key structures such as the obturator nerve, is imperative to success and progression to extended radical resection is usually required [24].

## 7. Palliation for Recurrent Rectal Cancer

In patients not fit for surgical intervention or with disease deemed unresectable, radiotherapy may play a role [37]. It is very effective in the treatment of pelvic pain and ongoing bleeding. The use of external beam radiotherapy has been reported to control pelvic pain in over 90% of cases and thus provide improved quality of life for affected patients. In patients with impending obstruction the use of self-expanding metal stents (SEMSs) is effective [38]. If the tumour is very low or the stent fails then a laparoscopic defunctioning stoma may be required to alleviate impending instruction. In patients with bilateral hydronephrosis ureteric stents either retrograde or antegrade will relieve the obstruction and prevent renal failure. If the ureters are completely obstructed then nephrostomy tubes are required.

## 8. Surgical Management: Multimodal Approach

While it is widely agreed that multimodal therapy is the future of management of recurrent rectal cancer, the use of Intraoperative Radiotherapy (IORT) remains controversial. IORT may be advantageous when bony involvement precludes the possibility of R0 resection [5]. Indeed, several studies have demonstrated a benefit in survival with IORT, particularly in combination with preoperative chemotherapy [12, 39]. IORT has been shown to result in significantly better three-year survival, disease-free survival, and local control in IORT-multimodal groups compared with historical control groups [40]. The overall survival after multimodal therapy at 5 years is approximately 30% at present [41] and when IORT is used as a component of this treatment, an increased survival rate of 15% can be demonstrated [39, 42].

## 9. The Future

Given the relatively poor prognosis despite multimodal treatment, the future of recurrent rectal cancer management lies in scientific progress, optimised technique, new treatments, and carefully designed clinical trials. The first case of transrectal high-intensity focused ultrasonography as a therapeutic option for advanced recurrent rectal cancer has recently been reported [43]. Potentially promising ongoing work includes identification of novel biomarkers as predictors of recurrence [19], discovery of novel alleles for use in targeted screening and personalized prevention [44–46], and development of systematic clinical algorithms [24]. Individualization of therapy in the future may be possible with next-generation genotyping platforms [47]. Although the popularisation of TME has resulted in decreased incidence of recurrence in rectal cancer, pelvic sidewall recurrence has increased, and tumours are less likely to be contained within defined compartments. Advances in imaging modalities and technical progress, however, have facilitated better selection of candidates for resection and substantially improved outcome as a result. Strategies for early detection require improvement and surgical techniques should be standardised. At present, best practice should include meticulous preoperative planning and adoption of a multimodal approach in centres of excellence with early involvement of a multidisciplinary team. A considerable amount of time must be spent counselling the patient and their family to facilitate thorough understanding of inherent risks and to ensure realistic expectations.
